# Effect of China’s maternal health policy on improving rural hospital delivery: Evidence from two cross-sectional surveys

**DOI:** 10.1038/s41598-018-29830-8

**Published:** 2018-08-17

**Authors:** Xiaojing Fan, Yongjian Xu, Martyn Stewart, Zhongliang Zhou, Shaonong Dang, Duolao Wang, Jianmin Gao

**Affiliations:** 1 Department of Epidemiology and Health Statistics, School of Public Health, Xi’an Jiaotong University Health Science Centre, Xi’an, P. R. China; 2 Department of Clinical Sciences, Liverpool School of Tropical Medicine, Liverpool, UK; 3 School of Public Policy and Administration, Xi’an Jiaotong University, Xi’an, P. R. China; 4 Department of Education and Training, Liverpool School of Tropical Medicine, Liverpool, UK

## Abstract

This population-based cross-sectional study aims to explore the effect of China’s Rural Hospital Delivery Subsidy (RHDS) policy on the utilization of women’s hospital delivery between rural and urban areas. A total of 2398 women were drawn from the Fourth and Fifth National Health Service Surveys, from the Shaanxi province. A generalized linear mixed model was used to analyze the influence of the RHDS policy on the hospital delivery rate. Concentration index and decomposition methods were used to explore the equity of hospital delivery utilization. Prior to introduction of the RHDS policy, the difference in hospital delivery rates was −0.09 (95% CL: −0.16, −0.01) between rural and urban women when adjusting the influence of socioeconomic factors on hospital delivery; after implementation of the policy, the difference was reduced to 0.02 (95% CL: −0.01, 0.06). The horizontal inequity index was reduced from 0.084 to 0.009 for rural women and from 0.070 to 0.011 for urban women. China’s Rural Hospital Delivery Subsidy policy had some positive effect on reducing the gap between rural and urban women’s hospital delivery rate and inequity. However, there is still a pro-rich inequity of hospital delivery utilization for both rural and urban women.

## Introduction

Maternal mortality rate (MMR) is an important indicator to evaluate the health status in developing countries^1^. The World Health Organization (WHO) defines maternal death as “the death of a woman during pregnancy or within a period of 42 days after the end of the pregnancy, regardless of the pregnancy duration or location, from any cause related to or aggravated by pregnancy or by measures related to it, but not from accidental or incidental causes”^2^. Every day, approximately 830 women die from preventable causes related to pregnancy and childbirth, 99% of which occur in developing countries^3^. In Brazil, MMR is 68 deaths per 100,000 livebirths^4^; In Bangladesh, MMR is 194 deaths per 100,000 livebirths^5^; in Ghana, it is 1004 per100,000 livebirths^6^; in southern Nigeria, it is 1908 per 100,000 livebirths^1^. In China, the MMR was 27 per 100,000 livebirths^7^, lower than Brazil, Bangladesh, Ghana and southern Nigeria, but still higher than developed countries (12 per 100,000 livebirths)^8^. Hospital delivery, where pregnant women give birth to babies in hospital, is promoted as an effective strategy to prevent maternal and perinatal mortality since it is recognized that most obstetric complications occur at the time of delivery and cannot be predicted^9^.

The Chinese government has made many efforts to improve hospital delivery rates since 1995, such as the “Safe motherhood” and “Reducing maternal mortality and eliminating newborn tetanus” policies^10,11^. In 2003, three basic medical insurance schemes called “Urban Employee Basic Medical Insurance scheme”, “New Rural Cooperative Medical Scheme” and “Urban Resident Basic Medical Insurance scheme” were introduced and can cover women’s hospital delivery costs partially^12^. China’s latest health system reform was initiated in April 2009 and one of its aims was to ensure the safety of maternal women. As part of this, a key policy, the Rural Hospital Delivery Subsidy (RHDS) policy for rural women was introduced in September 2009 to narrow the difference between maternal mortality rates existing between urban and rural areas. The RHDS policy is to reimburse hospital delivery fees for rural women, aiming to achieve more than a 95% hospital delivery rate^13^. It provides 500 Ren Min Bi for each rural woman delivering at hospital and it is combined with New Rural Cooperative Medical Scheme to ease the financial burden of rural hospital delivery^14^.

Up to now, there have been some published studies supporting the role of the RHDS policy in influencing hospital delivery. Yang *et al*. found the RHDS policy promoted the hospital delivery services and reduced the regional difference^14^; Shen *et al*. observed the rate of hospital delivery increased after implementation of the RHDS policy in western China^15^; as did the studies of Song *et al*.^16^ and Zhang *et al*. in Sichuan Province^17^. However, these study data are based on annual reports or local surveys at the level of the county. Whilst county-level reports are valuable, the relationship between RHDS policy and hospital delivery can only be fully understood at the individual level. The data through face-to-face interview can obtain the first-hand information directly, investigators can observe the respondent’s response in a timely manner to get a more complete and accurate information^18^. However, there is no data available collected through face-to-face interviews to explore the relationship between RHDS policy and hospital delivery. Shaanxi Province, in the west of China, is an appropriate study area because of it’s predominantly rural character and high proportion of poor in the population, the type of area where the policy is needed most. Determining whether this policy has a positive effect on hospital delivery is important local health government strategies to promote the safe and health of maternal and newborns. Equity in healthcare has been a long-term guiding principle of health policy, and inequity remains a major challenge for health care planners and policy makers all over the world. In this study, two representative surveys based on face-to-face interviews are used firstly to explore whether the RHDS policy has made some influence on improving the hospital delivery rate in Shaanxi Province and the specific magnitude of inequity on hospital delivery utilization changes. Finally, we draw preliminary conclusions about whether the Chinese maternal health policies are progressing in the right direction.

## Results

### Changes of hospital delivery rate

Table 1 presents the basic characteristics between urban women and rural women before and after the introduction of the RHDS policy. Before the policy, the difference in proportion between rural women who had a hospital delivery was 18.57% points (95% CL: 10.75%, 26.40%) lower than that of urban women [46.00% (95% CL: 41.32%, 50.67%) vs. 64.57% (95%CL: 58.30%, 70.85%); χ^2^ = 20.43, P < 0.001]. After the policy, the difference in rate was reduced to 1.47% (95% CL: 0, 3.98%) between rural and urban women [91.63% (95% CL: 89.94%, 93.33%) vs 93.10% (95%CL: 91.23%, 94.96%); χ^2^ = 1.26, *P* = 0.262]. Table 2 shows the results of the differences of Rural Hospital Delivery Subsidy policy on hospital delivery rate before and after RHDS policy by two GLMMs. When adjusting for the influence of women’s age, health score, prenatal visits, chronic disease, health insurance, annual personal expenditure, parity, education and work status on hospital delivery rate in model 2, the difference in hospital delivery rates was −0.09 (95%CL: −0.16, −0.01) between rural and urban women before the policy. After the policy, there was no difference in hospital delivery rates between them (mean difference: 0.02; 95%CL: −0.01, 0.06). This suggests the intervention of RHDS policy had some influence on reducing the gap in hospital delivery rate between rural and urban women.

**Table 1:** Descriptions of characteristics among participants before and after China’s Rural Hospital Delivery Subsidy [$\bar{x}$ ± *sd*/n (%)]. $\bar{x}$: mean; *sd*: Standard Deviation.

Variables	Before policy (n = 660)			After policy (n = 1738)		
	Urban	Rural	*P*	Urban	Rural	*P*
Hospital delivery						
No	79 (35.43)	236 (54.00)	<0.001	49 (6.9)	86 (8.37)	0.262
Yes	144 (64.57)	201 (46.00)		661 (93.10)	942 (91.63)	
Age (years)						
≤25	57 (25.56)	147 (33.64)	<0.001	195 (27.46)	350 (34.05)	<0.001
26–30	107 (47.98)	137 (31.35)		302 (42.54)	332 (32.30)	
≥31	59 (26.46)	153 (35.01)		213 (30.00)	346 (33.65)	
Health score	85.30 ± 8.59	85.41 ± 10.47	0.886	88.03 ± 8.25	86.98 ± 8.31	0.009
Chronic disease						
No	215 (96.41)	426 (97.48)	0.437	683 (96.20)	989 (96.21)	0.992
Yes	8 (3.59)	11 (2.52)		27 (3.80)	39 (3.79)	
Annual personal expenditure (Chinese Yuan)						
Poorest	54 (24.32)	249 (57.24)	<0.001	38 (5.35)	143 (13.91)	<0.001
Poorer	56 (25.23)	112 (25.75)		85 (11.97)	224 (21.79)	
Middle	55 (24.77)	48 (11.03)		130 (18.31)	245 (23.83)	
Richer	40 (18.02)	17 (3.91)		169 (23.80)	251 (24.42)	
Richest	17 (7.66)	9 (2.07)		288 (40.56)	165 (16.05)	
Parity						
1	193 (86.94)	258 (59.58)	<0.001	432 (60.93)	496 (48.39)	<0.001
≥2	29 (13.06)	175 (40.42)		277 (39.07)	529 (51.61)	
Prenatal visits	7.67 ± 4.16	5.06 ± 2.70	<0.001	6.36 ± 3.39	5.71 ± 2.56	<0.001
Education						
≤Primary school	8 (3.59)	133 (30.57)	<0.001	78 (11.02)	232 (22.59)	<0.001
Middle school	84 (37.67)	245 (56.32)		371 (52.40)	620 (60.37)	
≥High school	131 (58.74)	57 (13.10)		259 (36.58)	175 (17.04)	
Employment						
No	99 (44.39)	65 (14.87)	<0.001	220 (30.99)	156 (15.18)	<0.001
Yes	124 (55.61)	372 (85.13)		490 (69.01)	872 (84.82)	
Health insurance						
No	45 (20.18)	11 (2.52)	<0.001	87 (12.25)	27 (2.63)	<0.001
Yes	178 (79.82)	426 (97.48)		623 (87.75)	1001 (97.37)

**Table 2:** Rural-urban difference in hospital delivery before and after China’s Rural Hospital Delivery Subsidy policy (n = 2398).

Comparison	Model 1				Model 2			
	Mean difference	95% Confidence Limits		*P*	Mean difference	95% Confidence Limits		*P*
		Lower	Upper			Lower	Upper	
Before policy								
Rural vs Urban	−0.15	−0.22	−0.07	<0.001	−0.09	−0.16	−0.01	0.028
After policy								
Rural vs Urban	−0.02	−0.05	0.02	0.343	0.02	−0.01	0.06	0.230

Generalized linear mixed model was used to estimate the interaction effect between China’s Rural Hospital Delivery Subsidy policy and site on hospital delivery rate. In model 1, policy, site and policy*site are treated as fixed effects and village as random effect. The model 2 is the model 1 plus women’s age, health score, prenatal visits during pregnancy, chronic disease, health insurance, annual household income, parity, education and work.

### Equity of hospital delivery utilization

**Figure 1:**
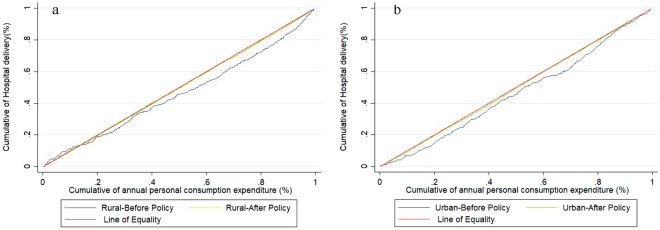
Concentration curves on hospital delivery before and after China’s Rural Hospital Delivery Subsidy policy (a = Rural, b = Urban).

Figure 1 shows that before the RHDS policy, concentration curves both in rural and urban women lay below the line of equality significantly, indicating that hospital delivery was more concentrated among the rich. However, the concentration curves were closer to the line of equality after the policy and the difference in concentration index between urban and rural women was reduced from 0.012 to −0.001 (Table 3). In addition, the concentration index of occurring hospital delivery in rural women decreased significantly (*P* = 0.020) from 0.082 (95% CL: 0.038, 0.156) to 0.009 (95% CL: −0.001, 0.022). This decreasing trend remains for urban women (*P* = 0.003): 0.070 (95% CL: 0.020, 0.172) before the policy, and after 0.010 (95% CL: −0.002, 0.024, Table 3). Therefore, the differences in inequality of hospital delivery were reduced after the policy but still favoring the rich both in rural and urban women.

**Table 3:** Inequality of urban and rural women’s hospital delivery before and after China’s Rural Hospital Delivery Subsidy policy (n = 2398).

Variable	Before policy			After policy		
	CI^1^	95% Confidence Limits		CI^1^	95% Confidence Limits	
Rural	0.082	0.038	0.156	0.009	−0.001	0.022
Urban	0.070	0.020	0.172	0.010	−0.002	0.024
Difference	0.012	—	—	−0.001	—	—

^1^CI: Concentration Index.

By decomposing the concentration index of the hospital delivery, the socioeconomic-related inequalities were decomposed into relative contributions of each determinant (see Supplementary Tables S1 and S2). Quantifying the corresponding contributions expressed as a percentage of each determinant before and after China’s Rural Hospital Delivery Subsidy policy, most of the socioeconomic inequality in hospital delivery was attributable to annual personal expenditure, at 73.39 and 90.84 for rural women, 61.42 and 85.31 for urban women. Finally, it was computed that horizontal inequity indexes of occurring hospital delivery after the policy are 0.009 for rural women and 0.011 for urban women, evidencing a pro-rich inequity of hospital delivery utilization. In addition, the difference in horizontal inequity indexes of hospital delivery utilization between rural and urban women reduced from 0.014 (0.084 vs 0.070) before the policy to −0.002 (0.009 vs 0.011) after the policy, indicating the inequity gap of hospital delivery utilization had been reduced after the policy (Table 4).

**Table 4:** Contributions of factors and horizontal inequity of women’s hospital delivery before and after China’s Rural Hospital Delivery Subsidy policy (n = 2398).

Groups	Rural				Urban			
	Before policy		After policy		Before policy		After policy	
	Contribution to CI^1^	%^2^	Contribution to CI^1^	%^2^	Contribution to CI^1^	%^2^	Contribution to CI^1^	%^2^
CI^1^								
	0.082	100	0.009	100	0.070	100	0.010	100
Contributions of factors to CI^1^								
Needs variables^3^	−0.002	−2.19	0.000	−5.14	0.000	−1.47	−0.001	−5.08
Annual personal expenditure (Chinese Yuan)								
	0.060	73.39	0.008	90.84	0.043	61.42	0.008	85.31
Other variables^4^	0.010	12.08	0.004	31.13	0.004	5.06	0.001	10.14
Residual	0.014	16.72	−0.003	−16.83	0.230	34.99	0.002	9.63
Horizontal inequity index								
	0.084	—	0.009	—	0.070	—	0.011	—

^1^Concentration Index. ^2^Pure percentage contributions of determinants to the socioeconomic inequality in hospital delivery. ^3^Including age, health score and chronic disease. ^4^Including women’s prenatal visits during pregnancy, health insurance, parity, education and work.

## Discussion

China bears some burden of global maternal mortality, the largest part being in the poor western provinces^19^. Hospital delivery should be completely covered because it has been recognized as an effective strategy in reducing maternal mortality. Although previous studies show women’s higher socioeconomic characteristics (including age, education, work or not, parity and so on) may increase the hospital delivery rate, there is still a gap between urban and rural women^20^. In this study, we focused on China’s Rural Hospital Delivery Subsidy policy and explored its influence on reducing the gap between rural and urban women’s hospital delivery rate when adjusting for their socioeconomic characteristics. According to these two representative samples of health service surveys, the evidence supports the positive effect of the policy in reducing the difference in hospital delivery rate between rural and urban women. This finding is consistent with the evaluation of RHDS policy studies made by Song *et al*., Zhang *et al*., Shen *et al*. and Yang *et al*.^14–17^. It is the first study based on two representative surveys to explore the relationship between RHDS policy and hospital delivery in Shaanxi Province. A positive direction of the RHDS policy in improving the safe and health of maternal and newborns has been concluded and provides a strong evidence base to inform health policy. Now, the hospital delivery rate for rural women is at a similar level to that of urban women (91.63% vs 93.10%) in Shaanxi Province. Besides, the hospital delivery rate for rural women in Shaanxi was 91.63% in 2013 which, whilst still higher than the rate of 86.3% reported by Liu *et al*. in 2011^21^, is still lower than the rates in Zhejiang (99.98%)^22^ and other regions (97.8%) in China^16^. It is higher than for many developing countries though. In Pakistan, the rate of hospital delivery was 40% for rural areas^23^ in 2012–2013, and among Indian rural women it was 69.7%, as reported through the Sample Registration System, Registrar General of India^24^. So, whilst the status of rural and urban hospital delivery rate in Shaanxi was not low, and it had increased in recent years.

China is a huge country with significant rural-urban differences, and rural areas are usually recognized as having a lower quality of health care^25^. The government conducted the health system reform and introduced a basic health insurance system in order to eliminate the rural-urban disparity in health care. These two policies helped redistribute health resources towards rural areas. However, substantial rural-urban inequality in delivery still exists with more hospital deliveries in urban than in rural areas^26,27^. One of the encouraging findings in this study is that the difference in rural hospital delivery inequality was also reduced after the introduction of the RHDS policy, demonstrating that implementation of maternal policies since 2009 has led the rural health service utilization development in a positive direction^28^. However, the concentration indexes of hospital delivery utilization between rural and urban were positive, showing that obvious pro-rich inequalities of hospital delivery utilization still remain, and indicating that a disproportionate share of hospital delivery resources is utilized by wealthier people despite lower need. These results are consistent with findings of many other studies in different parts of the world^26,29,30^. Therefore, the focus of public health policy still needs to remain on the expansion of the maternal health utilization with an emphasis among poor women.

The study has a few limitations. First, this is an observational study and the determinants of hospital delivery included in this study are limited by the pre-specified questions in the surveys. There could be some potential unobserved confounding factors we did not control for. Second, the imbalanced data before (660) and after (1738) the policy may have two potential impacts on the results and conclusions. One potential impact is the smaller data before the policy are less representative of the study population than that of the larger one after the policy, introducing possible more selection bias into the statistical results related to the data before the policy; another potential impact is the imbalanced data may generate results with less statistical efficiency (eg, larger standard error and less statistical significance) compared with the balanced one. Last but not the least, all the data were collected by a self-report approach, and there may be recall bias. However, it is suggested that, as pregnancy and childbirth are prominent life events, the associated heightened attention is likely to reduce effects of recall bias.

In summary, the evidence supports the positive effect of China’s Rural Hospital Delivery Subsidy policy in reducing the gap in hospital delivery rate and inequity between rural and urban women. However, there is still a pro-rich inequity of hospital delivery utilization for both rural and urban women and efforts should still be made to increase utilization of maternal health services in order to realize the full coverage of hospital delivery and eliminate the rural-urban inequity in health care.

## Methods

### Study setting and data source

Data were drawn from the 4^th^ (in 2008) and 5^th^ (in 2013) National Health Service Surveys (NHSS) conducted in Shaanxi Province before and after the introduction in 2009 of China’s Rural Hospital Delivery Subsidy (RHDS) policy. The NHSS is a national representative survey conducted by the National Health and Family Planning Commission of China every five years^31^. Considering the hierarchical structure of Chinese administrative districts and the imbalanced population distributions among the different provinces, a multistage stratified sampling method was used to ensure the samples were representative of the whole population of each province. The structured strategy for sampling in Shaanxi in the 4^th^ and 5^th^ NHSS was introduced by Zhou *et al*. and Yang *et al*.^32,33^.

During the survey, all household members were interviewed face to face individually using a structured household questionnaire (see Supplementary Questionnaire S1 and S2). A total of 18,290 household members in the 4^th^ NHSS and 57,529 household members in the 5^th^ NHSS were collected. For our study, only women who had at least one delivery were selected as the sampling unit of interest in the 4^th^ NHSS. From the 5^th^ NHSS, only women whose last delivery occurred after January 2010 were selected considering the publishing time of RHDS policy (September 2009) and the consistencies in implementation among hospitals and related departments (for example, the health insurance department). Finally, data from 660 women in the 4^th^ NHSS and 1,738 women in the 5^th^ NHSS were utilized for this analysis. Given the hospital delivery rate (88.8%) in rural area of China before the 4^th^ National Health Services Survey^34^, the sample size was calculated as 510 with a permissible error of 3%, a type I error of 0.05 and an expected 20% non-response rate. Thus, the number of the women from these two cross-sectional studies met the requirement of hospital delivery analysis.

### Ethics

This study protocol was approved by the Ethics Committee of Xi’an Jiaotong University Health Science Center (the 4^th^ NHSS Approval No. 2014–204 and the 5^th^ NHSS Approval No. 2015-644). In addition, the study was in accordance with the 1964 Helsinki Declaration and its later amendments or comparable ethical standards. Verbal informed consent was obtained by NHSS surveyors from each participant before the original survey.

### Main outcome and predictor variables

In the household questionnaire of the 4^th^ and 5^th^ NHSS, women were asked to describe the type of delivery institution from six category options: county hospital and above, maternal and child health care institution, township health center, community health center, village clinic and at home. According to China’s status for medical institutions after December 2009, we divided them into two groups: ≥secondary institution (including county hospital and above, maternal and child health care institution) and ≤primary institution (including township health center, community health center, village clinic and at home). Women who had a delivery at ≥secondary institution were identified as having hospital delivery. The settings characterized as primary institutions were considered to lack sufficient equipment, doctors and, or nurses to ensure a safe delivery. The indicator used to measure the effect of the Rural Hospital Delivery Subsidy policy for rural maternal women was the occurrence of hospital delivery (a binary outcome variable). According to the RHDS policy that only rural women could receive a subsidy after a hospital delivery, rural women were selected to be the intervention group. The policy had not yet been implemented among urban women from the 4^th^ NHSS to the 5^th^ NHSS, making the urban women to be an excellent control for this study. It is hypothesized that the occurrence of hospital delivery in rural areas would be increased after implementing the RHDS.

### Statistical analysis

Generalized linear mixed models (GLMM) are an extension of generalized linear models and include both fixed and random effects^35^. The response variable can come from different distributions. In this study, GLMM was employed to analyze the effect of the RHDS policy on hospital delivery after controlling a number of confounding factors. These variables were selected based on previous studies but constrained by the variables collected in the survey^16,36,37^. The characteristics of the whole variables used are shown in Table 1. The variables policy, site, and interaction between policy and site were specified as fixed effects, and the village where women lived as a random effect. Health insurance, health score, annual personal expenditure, parity, chronic disease, prenatal visits, age, education, work were covariates. In a GLMM, the linear prediction *η* is the combination of the fixed and random effects excluding the residuals.

1 $$\begin{array}{rcl}\eta  & = & X{\rm{\beta }}+Z{\rm{\gamma }}\\ g(\,\cdot \,) & = & {\rm{link}}\,{\rm{function}}\\ h(\,\cdot \,) & = & {g}_{-1}(\,\cdot \,)={\rm{inverse}}\,{\rm{link}}\,{\rm{function}}\end{array}$$

*g*(⋅) is the link function which relates the outcome y to the linear predictor *η*. The equation is as follow:

2 $$g(E(Y))=X{\rm{\beta }}+Z{\rm{\gamma }}$$

Here, we use the multiple generalized linear mixed model:

3 $$\eta ({y}_{ij})={\beta }_{0j}+{\beta }_{1j}{x}_{1i}+{\beta }_{2j}{x}_{2i}+{\beta }_{3j}{x}_{1i}{x}_{2i}\cdots +{\beta }_{pj}{x}_{pi}+{\varepsilon }_{ij}$$

In equation (3), *y*_*ij*_ is the hospital delivery takes the value of 0 and 1. *β*_0,*j*_ is a constant, *β*_*pj*_ represent the effects of *x*_*pi*_ on *y*, and *ε*_*ij*_ is a random error. The link function is identity. In this study, interaction effect may exist between RHDS policy and site (urban, rural) when *β*_3,*j*_ ≠ 0^38^.

### Methods to measure hospital delivery inequality

Concentration index (CI) was employed to measure the extent of income-related inequality of healthcare utilization^39^. It is defined as twice the area between the concentration curve and the line of equality and ranges from −1 to +1^40^. The formula for computing the concentration index is as follows:

4 $$C=\frac{2}{\mu }{co}{{v}}_{w}({{\rm{y}}}_{{i}},{R}_{i})$$

where *C* stands for concentration index, *y*_*i*_ is the hospital delivery status of the *i*th individual, and *R*_*i*_ is the fractional rank of the *i*th individual (for weighted data) in terms of the index of annual personal economic status. *μ* is the (weighted) mean of hospital delivery index and *cov*_*w*_ denotes the weighted covariance. If *C* is positive, it means high-income people utilize more hospital delivery than their low-income counterparts. Meanwhile, the *C* is negative if the low-income group utilizes more hospital delivery than their rich counterparts. When all hospital delivery resources are utilized by low-income group the concentration index will be −1 and it will be +1 when the high-income group are favored in hospital delivery utilization. Hospital delivery is equitably utilized by the poor and the rich when the index is 0^41^.

### Horizontal inequity of hospital delivery

Horizontal inequity index (HI) is a summary measure of the magnitude of inequity in the dependent variable, used to measure whether the extent of deviation in the use of healthcare for people is equal for healthcare irrespective of their income^42,43^. HI is computed by subtracting the contribution of need variables (such as age, health score and having chronic disease or not) from the concentration index of hospital delivery, which is used to measure the equity of hospital delivery. Decomposition methods enable researchers to quantify each determinant’s true contribution to measured income-related inequality with the controlling of other determinants^44^. Since the outcome variable, hospital delivery, was binary with the range of (0, 1), Probit regression model was used to indirectly standardize the healthcare service utilization. As the standardization of health utilization holds for a linear model of healthcare, we applied the linear approximation to the Probit model to extract marginal effects of each determinant on observed probabilities of the outcome variable. The formula can be written as follows^39,45^:

5 $${y}_{i}=G(\alpha +\sum _{j}{\beta }_{j}{x}_{ji}+\sum _{k}{\gamma }_{k}{z}_{ki})+{\varepsilon }_{i}$$

*G* is functional transformation, *y* is the dependent variable, *x*_*ji*_ are needs variables, and *z*_*ki*_ are control variables. Then the standardized need was estimated using the following equation:

6 $${\hat{y}}_{i}^{IS}={y}_{i}-G(\hat{{\rm{\alpha }}}+\sum _{j}{\hat{\beta }}_{j}{x}_{ji}+\sum _{k}{\hat{\gamma }}_{k}{\bar{z}}_{k})+\frac{1}{n}\times \sum _{i=1}^{n}G(\hat{{\rm{\alpha }}}+\sum _{j}{\hat{\beta }}_{j}{x}_{ji}+\sum _{k}{\hat{\gamma }}_{k}{\bar{z}}_{k})$$

where ${\hat{y}}_{i}^{IS}$ is standardized hospital delivery utilization, *n* is sample size. The more hospital delivery allocated to the needed, the less inequity of hospital delivery utilization.

All questionnaires had been checked for missing data and outliers, then cleaned prior to data analysis. Continuous variables were summarized as means with standard deviations, and categorical variables as number and percentages. Differences in variables between rural and urban areas were compared by either *t* test or chi-squared test. The statistical analyses were performed using SAS 9.4 (SAS Institute, Cary, NC, USA) and STATA statistical software version 12.0 (StataCorp LP, College station 77845, USA). A two-tailed *P* value < 0.05 was considered statistically significant.

### Availability of materials and data

The datasets analyzed during the current study are not publicly available due to confidential data only be available from the Shaanxi Health and Family Planning Commission for researchers who meet the criteria but are available from the corresponding author on reasonable request. Researchers who want to use these data should contact Jianmin Gao (gaojm@mail.xjtu.edu.cn).

## Electronic supplementary material

Supplementary information
