# Supplementary material for: Effect of China’s maternal health policy on improving rural hospital delivery: Evidence from two cross-sectional surveys
Source: Sci Rep. 2018 Aug 17;8:12326. doi: 10.1038/s41598-018-29830-8 (PMC6098125; doi:10.1038/s41598-018-29830-8)
Supplement: Supplementary file 1 — Supplementary information [file 41598_2018_29830_MOESM1_ESM.pdf]

Effect of China's maternal health policy on improving rural hospital delivery: Evidence from two cross-sectional surveys

Xiaojing Fan[1,2], Yongjian Xu[3], Martyn Stewart[4], Zhongliang Zhou[3], Shaonong Dang[1], Duolao Wang[2, *], Jianmin Gao[3,*]

Supplementary Table S1 Decomposition analysis of concentration index on rural women's hospital delivery before and after China's Rural Hospital Delivery Subsidy policy (n=1465)

Variables	Before policy (n=437)				After policy (n=1028)			
	Elasticity	CI [1]	Contribution to CI[1]	%[2]	Elasticity	CI [1]	Contribution to CI[1]	%[2]
Age (years)								
≤25	Reference				Reference			
26-30	0.068	0.053	0.004	4.35	0.007	-0.018	0.000	-1.44
≥31	0.158	-0.029	-0.005	-5.50	0.010	-0.023	0.000	-2.68
Health score	0.898	-0.001	-0.001	-1.60	-0.128	0.002	0.000	-2.43
Chronic disease								
No	Reference				Reference			
Yes	0.013	0.036	0.000	0.56	-0.001	-0.091	0.000	1.41
Annual personal expenditure (Chinese Yuan)								
Poor	Reference				Reference			
Poorer	0.025	0.284	0.007	8.49	0.000	-0.406	0.000	0.82
Middle	0.029	0.675	0.019	23.60	-0.001	-0.072	0.000	0.59
Richer	0.017	0.874	0.015	18.52	-0.003	0.331	-0.001	-13.24
Richest	0.018	1.064	0.019	22.78	0.012	0.757	0.009	102.67
Parity								
1	Reference				Reference			
≥2	-0.468	-0.006	0.003	3.36	-0.071	-0.004	0.000	3.39
Prenatal visits	0.107	0.014	0.001	1.79	0.058	0.027	0.002	18.31
Education								
≤Primary school	Reference				Reference			
Middle school	0.110	0.034	0.004	4.54	0.036	-0.008	0.000	-3.20
≥High school	0.024	0.115	0.003	3.40	0.010	0.164	0.002	18.10
Employment								
No	Reference				Reference			
Yes	-0.188	0.005	-0.001	-1.13	-0.018	0.001	0.000	-0.27
Health insurance								
No	Reference				Reference			
Yes	0.423	0.000	0.000	0.12	0.079	-0.006	0.000	-5.20

[1] CI: Concentration Index; [2] Pure percentage contributions of determinants to the socioeconomic inequality in hospital delivery.

Supplementary Table S2 Decomposition analysis of concentration index on urban women's hospital delivery before and after China's Rural Hospital Delivery Subsidy policy (n=933)

Variables	Before policy (n=223)				After policy (n=710)			
	Elasticity	CI [1]	Contribution to CI[1]	%[2]	Elasticity	CI [1]	Contribution to CI[1]	%[2]
Age (years)								
≤25	Reference				Reference			
26-30	-0.036	0.058	-0.002	-3.02	0.003	0.033	0.000	0.96
≥31	-0.029	0.015	0.000	-0.62	-0.001	-0.040	0.000	0.61
Health score	0.334	0.005	0.002	2.24	-0.164	0.003	-0.001	-5.39
Chronic disease								
No	Reference				Reference			
Yes	-0.001	0.032	0.000	-0.07	0.001	-0.204	0.000	-1.26
Annual personal expenditure (Chinese Yuan)								
Poor	Reference				Reference			
Poorer	0.072	-0.075	-0.005	-7.65	0.007	-0.768	-0.005	-52.63
Middle	0.018	0.281	0.005	7.38	0.005	-0.361	-0.002	-17.88
Richer	0.052	0.450	0.023	33.37	0.016	0.067	0.001	11.03
Richest	0.036	0.547	0.020	28.32	0.029	0.477	0.014	144.79
Parity								
1	Reference				Reference			
≥2	-0.076	-0.028	0.002	3.05	-0.024	-0.016	0.000	3.89
Prenatal visits	-0.198	0.047	-0.009	-13.33	0.005	0.000	0.000	0.02
Education								
≤Primary school	Reference				Reference			
Middle school	-0.059	-0.235	0.014	19.85	-0.012	-0.077	0.001	9.55
≥High school	0.048	0.177	0.008	12.09	0.007	0.143	0.001	10.52
Employment								
No	Reference				Reference			
Yes	-0.062	-0.042	0.003	3.69	-0.026	-0.004	0.000	1.12
Health insurance								
No	Reference				Reference			
Yes	0.182	-0.078	-0.014	-20.29	0.041	-0.035	-0.001	-14.96

[1] CI: Concentration Index; [2] Pure percentage contributions of determinants to the socioeconomic inequality in hospital delivery.

Supplementary Questionnaire S1 The household questionnaire of the fourth National Health Service Survey

Household Questionnaire

The National Health Service Survey in 2008

Health's ministry of the People's Republic of China

June 2008

The Declaration of Household Survey's investigator

Dear residents,

Many greetings!

We are the investigators from the Fourth National Health Service Survey. This investigation is organized by the National Health Administration Department and is approved by the National Bureau of Statistics. The purpose of the National Health Service Survey is to understand the residents' healthy status and provide the basic reference to the health policy makers. According to the National statistical law of the People's Republic of China's relevant regulations, the whole questions we investigated is promised to be secrets and only be used for statistical analyses. We hope you can answer the following questions honestly. Thank you very much for your support and cooperation!

This survey is a single survey of private families; information shall not be divulged without consent" 《National statistical law of The People's Republic of China》(The third chapter, the fourteenth article)

Table No.: National Health Survey 1 Table
Establishment department: National Health's ministry
Approval authority: National Bureau of Statistics
Approval number: National Bureau of Statistics draft [2008]No.18

The Home Page of the Household Questionnaire

Name of householder: _______________ telephone number: _______________

Family Address: ________County (City / district) ________Town (Street)

_______Village (Neighborhood committee) ____________ (Detailed address)

County (City / district) Administrative area No.□□□□□□

Town (Street) area No. □ Village (Neighborhood committee) area No. □

Resident code □□

When you have finished your investigation, fill in the following form (the result is filled in the right space)

The number of resident within 6 months:	
Including: married women aged 15-49 years old	
children aged ≤ 5 years old	
aging people aged ≥ 60 years old	
The number of people who had been injured within the first two weeks of the survey	
The number of people who had been out of hospital within 12 months before the survey	
(In rural area)The number of people who work outside their hometown and their accompanying dependents	
The type of this household: (1) Selected (2) Substituted	

The end date of Investigation: ____________ Investigator (Signature):

Verification date: ____________ Investigation Instructor (Signature):

Table I Questionnaire of Family's Basic Characteristics

One family member who is familiar with the family's basic characteristics was asked to answer the questions in table 1

No.	Questions and options	Answer
1	How many people are there in your family? (Including the people in your household register, relatives, housekeeper and so on?	
2.	How many people who are at home during 6 months?	
3	(In the rural area) How many people are there working outside the county town?(including their spouse, children, parents and so on)	
4	What type of house do you have? (1) Building (2) Brick bungalow (3) Adobe bungalow (4) Others	
5	How many square meters is your house? (m^2)	
6	How many televisions do you have?	
7	What's the type of your telephone? (1) Fixed telephone (2) Mobil phone (3) Both of them (4) None	
8	What type of drinking water do you have? (1) Running water (2) Hand press well water (3) Protected well water (4) Collected Rainwater (5) Protected spring water (6) Unprotected well water (7) Unprotected spring water (8) Water from truck or trolley (9) Surface water (10) Canned water and the above 1-5 are used for cooking and washing (11) Canned water and the above 6-9 are used for cooking and washing (12)Others	
9	What type of washroom do you have? (1) Complete the sewer flushing (2) Urine diversion (3) Triplex methane (4) Funnel type (5) Three compartment septic tank (6) Double pit alternation (7) Ventilation type (8) Attic type (9) Pit antifreeze (10) Pit type toilet with cover plate (11) Pit type toilet without cover plate (12) manure bucket (13) Facility free / field (14) Others	
10	How many kilometers is the nearest medical institution from your home?<br>(1) < 1km (2) 1- (3) 2- (4) 3- (5) 4- (6) ≥ 5	
11	How many minutes does it take from your home to the nearest medical institution?<br>(The fastest way to get on foot or by transport)	

No.	Questions and options	Answer
12	How much was the total household income in the last year? (RMB) (Urban households are disposable income; rural households are net income)	
13	How much was the total household consumption expenditure in the last year? (RMB)	
14	Including: How much was the food expenditure?	
15	How much was the daily living expenditure?	
16	How much was the transportation and communication expenditure?	
17	How much was the housing, water, electricity and fuel expenditure	
18	How much was the education expenditure	
19	How much was the entertainment expenditure	
20	How much was the health care expenditure	
21	How much was the Others expenditure?	
22	How much is the tuition for children outside their hometown? (RMB)	
23	Last year, how much was it equivalent to RMB that your family enjoyed any kind of subsidy from the state or the collective? (Fill in 0 if there is none)	
24	Is your home identified as a government target for medical assistance? (1) Yes (2) No (3) I don't know	
25	Is your household listed as a local poverty-stricken or low-income household? (1) Yes (2) No (3) Both of them (4) None	
26	If yes, what is the most important reason leading to the poverty-stricken household or low-income household? (1) Small size of labor (2) Poor natural condition/disaster(3) Labor affected by disease (4) disease (5)Unemployment (6) human factor (7) Others	

Table II Questionnaire of Family member's Basic Characteristics

**Investigated members' code** (01 represent the householder, Other family members are numbered in accordance with the survey order. Once the code is determined, it cannot be changed.)		01	02	03	04	05	06
**A. Family member's basic information**							
1	Member's name: (01 is the householder)						
2	The relationship between the member and householder: (1)The householder (2) Spouse (3)Children (4) Grandchildren (5)Parents (6) Grandparent (7)Brothers/Sisters(8)Others						
3	Who will answer the following survey questions (**Investigators judge by themselves**)? (1)By oneself (2) Reply by others						
4	The reason of answering by others: (1) This family member is out (2) This family member is too young (3) This family member is in a insobriety (4) This family member is unwilling to answer (5)Others						
5	Sex: (1) Male (2) Female						
6	Ethnical: (1)Han (2) Mongol (3)Hui (4) Tibet (5) Uygurs (6) Miao (7) Others						
7	Birth Date: Year ________ (fill in four numbers, for example: 1998)						
8	Month ________						
**Continue to ask the members aged more than 15 years old** (member who were born before June, 1993)**, the other members skip to question 13**							
9	Marital status: (1) Single (2) Married (3) Widowed (4) Divorce (5) Others						

10	Education:<br>(1) Illiteracy (2) Primary school (3) Middle school (4) High school/ Technical school (5) Secondary specialized school (6) Junior College (7) undergraduate course and above						
11	Employment status:<br>(1) Employed (2) Retirement (3) Student at school (4)Out of work/Unemployment						
**Investigated members' code**		01	02	03	04	05	06
12	Type of work: (1) Person in charge of enterprise and institution (2) Professional and technical personnel (3) Administrative staff (4) Business / service personnel (5) Individual industrial and commercial households (6) Non-Agricultural production personnel (7)Non-farm worker (8) Agricultural laborers(Agricultural and forestry, fishery and water conservancy production personnel) (9)Others						
13	What type of medical insurance scheme do you have? (1) Urban Employee Basic Medical Insurance (2) Free medical care (3) Urban Resident Basic Medical Insurance (4) New Cooperative of Rural Medical Insurance (5) Others social medical Insurance (6)Don't attend						
14	How much do you pay if you attend urban resident basic medical insurance or new cooperative of rural medical insurance? (RMB)						
15	Have you purchased commercial medical insurance? (1) Yes (2) No						
16	How much do you pay if you purchase commercial medical insurance? (RMB)						

Table III Questionnaire of women aged 15-64 years old

The women who were born from August 16th 1949 to August 15th 1998 answer the following questions

**Investigated members' code (the code is the same as table 2)**		01	02	03	04	05	06
1	Have you had a gynecological examination in the past 12 months (breast, uterus and so on)?<br>(1) Yes (2) No						
2	How many times have you been pregnant? (Including abortion)						
3	How many children have you had? (Including the children that had dead)						
4	Have you had any child who was unfortunately died since 2003?<br>(1) Yes (2) No (Skip to question 10)						
5	If yes, how many?						
6	The date of the first child who was dead: (1)Jan-Jun 2003 (2)Jul-Dec 2003 (3) 2004<br>(4) 2005 (5) 2006 (6) 2007 (7) 2008						
7	The age of the dead child? (1)less than 1 years old (2) 1 years old (3) 2 years old<br>(4) 3 years old (5) 4 years old (6) more than 5 years old						
8	The date of the second child who was dead: (1)Jan-Jun 2003 (2)Jul-Dec 2003 (3) 2004<br>(4) 2005 (5) 2006 (6) 2007 (7) 2008						
9	The age of the dead child?<br>(1)less than 1 years old (2) 1 years old (3) 2 years old<br>(4) 3 years old (5) 4 years old (6) more than 5 years old						
10	(In the rural area) Does your husband work long distances outside your hometown?<br>(1) Yes (2) No						

Investigated members' code		01	02	03	04	05	06
11	(In the rural area) Have you got medical services provided by female doctors when you demand? (1) Yes (2) No (3) Never demand (4) I don't know						
12	Do you give a birth since Jan 1st 2003?<br>(1) Yes (2) No (The investigation is end)						
All of the following questions are asked about the status of the last child and his/her mother during pregnancy							
13	The last birth date of a child: Year ________						
14	Month ________						
15	The sex of your latest child?<br>(1)Male (2)Female						
16	How many times of prenatal visits have you had? (if one have no prenatal visit, fill in 0 and skip to the question 20)						
17	When did the first time of prenatal visit? (week)						
18	Where did you do the prenatal visits? (not more than 3 options)<br>(1) County Hospital and above (2) County Chinese Hospital and above (3)Maternal and child health care institution (4)Township Health Center (5) Community Health Centre<br>(6) Family planning guidance Station (7) Village clinic (8)At home						
19	During the prenatal visit, did the doctor do the following examination?						
19.1	Measure weight: (1) Yes (2) No						
19.2	Do blood test: (1) Yes (2) No						
19.3	Measure blood pressure: (1) Yes (2) No						

**Investigated members' code**		01	02	03	04	05	06
19.4	Urine test : (1) Yes (2) No						
19.5	B-ultrasonic examination: (1) Yes (2) No						
20	What was the gestational week? (week)						
21	What was the delivery way? (1) Vaginal (2) Vaginal Midwifery (3) Caesarean section						
22	What was the delivery institution?<br>(1) County Hospital and above (2) County Chinese Hospital and above (3)Maternal and child health care institution (4)Township Health Center (5) Community Health Centre (6) Family planning guidance Station (7) Village clinic (8)At home (9)Others						
23	What was the reason gave birth at home? (1)there is no need to hospital (2) There's not enough time (3)lack of money (4) Inconvenient transportation (5)Others						
24	Who was the midwife if women gave birth at home?(1) Doctor from township and above (2) Village doctor (3) Midwife (4) Part-time delivery personnel (5) Family member (6) Others						
25	What was the weight of newborn? (g)						
26	What was the total cost of delivery? (RMB)						
27	How much was reimbursement? (Fill in 0 If there was no)						
28	How many times of postnatal visits did you receive during 42 days after delivery? (Fill in 0 If there was no)						
29	How long did you first breastfeed your baby?<br>(1)Half an hour after born (2)between half an hour and an hour after born (3) within 24 hours (4)after 24hours (5) Never						

Supplementary Questionnaire S2 The household questionnaire of the fifth National Health Service Survey

Table No.: National Health Survey 1 Table
Establishment department: National Health and Family Planning Commission
Approval authority: National Bureau of Statistics
Approval number: National Bureau of Statistics draft [2013]No.65
Expiration date: December 2013

Household Questionnaire-The National Health Service Survey in 2013

Family Address: _____County(City / district)_____Town(Street)_____Village(Neighborhood committee)_________________(Detailed address)

Name of householder: ____________________ telephone number: _____________________

County(City / district) Administrative area No.□□□□□□ Town(Street) area No. □□□ Village(Neighborhood committee) area No. □□□

Resident code □□

The start date of Investigation: _______________ _____:_____

The end date of Investigation: ________________ _____:_____ Investigator (Signature):

Verification date: _____________ Investigation Instructor (Signature):

The Declaration of Household Survey's investigator

Dear residents,

Many greetings!

We are the investigators from the Fourth National Health Service Survey. This investigation is organized by the National Health Administration Department and is approved by the National Bureau of Statistics. The purpose of the National Health Service Survey is to understand the residents' healthy status and utilization of health care, to provide the basic reference to the health policy makers and improve the level of residents' health. According to the National statistical law of The People's Republic of China's relevant regulations, the whole personal information we investigated is promised to be secrets and only be used for statistical analyses. We hope you can answer the following questions honestly. Thank you very much for your support and cooperation!

"Anyone or any departments are not allowed to leak out the direct or indirect personal information which were got in statistical investigation to others. This information can only be used for statistical analyses"

《National statistical law of The People's Republic of China》(The third chapter, the twenty-fifth article)

The Detailed List of Survey

Table I Questionnaire of Family’s Basic Characteristics

Table II Questionnaire of Family Member’s Basic Characteristics

……

Table VI Questionnaire of women aged 15-64 years old

Table I Questionnaire of Family's Basic Characteristics

One family member who is familiar with the family's basic characteristics was asked to answer the questions in table 1

No.	Questions and options	Answer
1	How many people are there in the household register?	
2.	How many people who are in the household register are at home during 6 months?	
3	How many people are there at home during 6 months but not in the household register? (including relatives, housekeeper and so on)	
4	(In the rural area) How many household register people are there working outside the countytown?(including their spouse, children, parents and so on)	
5	How many kilometers is the nearest medical institution from your home? (1) < 1km (2) 1- (3) 2- (4) 3- (5) 4- (6) ≥ 5	
6	How many minutes does it take from your home to the nearest medical institution? (The fastest way to get on foot or by transport)	
7	What kind of medical institution do you usually go to for general diseases: (1) village clinic (2) Community Health Station (3) Township Health Center (4) Community Health Centre (5) comprehensive hospital (6) Chinese medicine hospital (7) Others	
8	Compared with 5 years age, how has your family changed in the convenience of visiting a doctor? (1) Substantially improved (2) Slight improved (3) unchanged (4) Slightly worse (5) Substantially worse	
9	Compared with 5 years age, how has your family changed in the cost of visiting a doctor? (1) Substantially decreased (2) Slight decreased (3) unchanged (4) Slightly increased (5) Substantially increased	
10	What kind of relationship do you think doctors and patients are most similar to? (1) Parents and children (2) Teachers and students (3) Friends (4) Work partner (5) Army friends (6) Superior and subordinate (7) Trading services (8) Others	
11	What is the most frequently used fuel in your cooking? (1) electricity (2) Coal gas/Natural gas/Petroleum gas (3) Methane gas (4) Coal oil (5) Coal (6) Firewood (7) Others	
12	What type of drinking water do you have? (1) Running water (2) Hand press well water (3) Protected well water (4) Collected Rainwater (5) Protected spring water (6) Unprotected well water (7) Unprotected spring water (8) Water from truck or trolley (9) Surface water (10) Others	

13	What type of washroom do you have? (1) Complete the sewer flushing (2) Urine diversion (3) Triplex methane (4) Funnel type (5) Three compartment septic tank (6) Double pit alternation (7) Ventilation type (8) Attic type (9) Pit antifreeze (10) Pit type toilet with cover plate (11) Pit type toilet without cover plate (12) manure bucket (13) Facility free / field (14) Others	
14	What type of house do you have? (1) Building (2) Brick bungalow (3) Adobe bungalow (4) Others	
15	How many square meters is your house? (m^2)	
16	How much was the total household income in the last year? (RMB) (Urban households are disposable income; rural households are net income)	
17	How much was the total household consumption expenditure in the last year? (RMB)	
18	Including: How much was the food expenditure?	
19	How much was the daily living expenditure?	
20	How much was the transportation and communication expenditure?	
21	How much was the housing, water, electricity and fuel expenditure	
22	How much was the education expenditure	
23	How much was the entertainment expenditure	
24	Including: How much was the health care expenditure	
25	How much was the Others expenditure?	
26	Is your household listed as a local poverty-stricken household? (1) Yes (2) No	
27	Is your household listed as a local low-income household? (1) Yes (2) No	
28	What is the most important reason leading to the poverty-stricken household or low-income household? (1) Small size of labor (2) Poor natural condition/disaster(3) Labor affected by disease (4) disease (5)Unemployment (6) human factor (7) Others	

Table II Questionnaire of Family member's Basic Characteristics

**A. Family member's basic information**							
**Investigated members' code** (01 represent the householder, Other family members are numbered in accordance with the survey order. Once the code is determined, it cannot be changed.)		01 (householder)	02	03	04	05	06
29	Member's name: (01 is the householder)						
30	The relationship between the member and householder: (1)The householder (2) Spouse (3)Children (4) Son-in-law/ Daughter-in-law (5)Parents (6) Wife's parents/Husband's parents (7) Grandparent (8) Grandchildren (9)Brothers/Sisters (10) Domestic service personnel (11)Others						
31	Who will answer the following survey questions (**Investigators judge by themselves**)? (1)By oneself (2) Reply by others						
32	household registration place: (1) County/District (2) Other County/District (3) Other province (4) Undetermined household registration place						
33	The type of household registration: (1) Agriculture (2) None-agriculture						
34	Sex: (1) Male (2) Female						
35	Ethnical: (1)Han (2)Zhuang (3)Hui (4) Uygurs (5) Mongol (6)Tibet (7) Manchu (8) Miao (9)Others						
36	Birth Date: Year ________ (fill in four numbers, for example: 1998)						
37	Month ________ (fill in two numbers, for example: 07)						
38	What is your height? (cm)						

Investigated members' code		01	02	03	04	05	06
39	What is your weight? (kg)						
40	Have you attended the Urban Employee Basic Medical Insurance scheme? (1) Yes (2) No						
41	Have you attended the Urban Resident Basic Medical Insurance scheme? (1) Yes (2) No						
42	Have you attended the New Cooperative of Rural Medical Insurance scheme? (1) Yes (2) No						
43	Have you attended the cooperative medical insurance for urban and rural residents? (1) Yes (2) No						
44	Have you purchased commercial medical insurance? (1) Yes (2) No						
45	Have you attended the other medical insurance? (1) Yes (2) No						
46	Are you the government's target of medical aid? (1) Yes (2) No (3) I don't known						
**Continue to ask the members aged more than 15 years old** (member who were born before August 15th, 1998)**, the other members skip to Table 3**							
47	Marital status: (1) Single (2) Married (3) Widowed (4) Divorce (5) Others						
48	Education: (1) Illiteracy (2) Primary school (3) Middle school (4) High school (5) Technical school (6) Secondary specialized school (7) Junior College (8) undergraduate course and above						
49	Employment status: (1) Employed (2) Retirement (3) Student at school (4)Out of work (5) Unemployment						
50	Type of work: (1) Person in charge of enterprise and institution (2) Professional and technical personnel (3) Administrative staff (4) Business / service personnel (5) Agricultural and forestry, fishery and water conservancy production personnel (6)Operators of production and transportation equipment (7) Soldier (8)Others						

Table VI Questionnaire of women aged 15-64 years old

The women who were born from August 16th 1949 to August 15th 1998 answer the following questions

**Investigated members' code**		01	02	03	04	05	06
167	Have you had a gynecological examination in the past 12 months? (1) Yes (2) No						
168	Have you had a smear test in the past 12 months? (1) Yes (2) No						
169	Have you had a breast examination in the past 12 months? (1) Yes (2) No						
170	(In the rural area)Have you got medical services provided by female doctors when you demand? (1) Yes (2) No (3) Never demand (4) I don't known						
171	(In the rural area) Does your husband work long distances outside your hometown? (1) Yes (2) No						
172	How many times have you been pregnant? (If women have never been pregnant, fill in 0 and stop this investigation)						
173	How many children have you had? (If women have never a child, fill in 0 and stop this investigation)						
174	The latest delivery date: Year ________ (fill in four numbers, for example: 1998)						
175	Month ________ (fill in two numbers, for example: 07)						
**Women who gave a birth since August 15th 2008 answer the following questions**							
176	The sex of your latest child? (1)Male (2)Female						
177	How many times of prenatal visits have you had? (if one have no prenatal visit, fill in 0 and skip to the next question)						

Investigated members' code		01	02	03	04	05	06
178	Did you have a blood test during the prenatal visits? (1) Yes (2) No						
179	Did you measure your blood pressure during the prenatal visits? (1) Yes (2) No						
180	Did you have a urine test during the prenatal visits? (1) Yes (2) No						
181	Did you have a B-ultrasonic examination during the prenatal visits? (1) Yes (2) No						
182	What was the delivery way? (1) Vaginal (2) Caesarean						
183	Who proposed the caesarean section?						
184	What was the delivery institution?<br>(1) County Hospital and above (2) Maternal and child health care institution (3) Township Health Center (4) Community Health Centre (5) Village clinic (6) At home						
185	Who was the midwife if women gave birth at home?<br>(1) Doctor from township and above (2) Village doctor (3) Midwife (4) Part-time delivery personnel (5) Family member (6) Others						
186	What was the weight of newborn? (g)						
187	What was the total cost of delivery? (RMB)						
188	How much did you pay for it? (Fill in 0 If there was no)<br>(Expenses from reimbursement and personal medical accounts are excluded)						
189	How many times of postnatal visits did you receive during 42 days after delivery? (Fill in 0 If there was no)						

When you have finished your investigation, fill in the following form (the result is filled in the right space)

1	The number of resident within 6 months:	
2	Including: women aged 15-49 years old	
3	children aged ≤ 5 years old	
4	The number of people who had been injured within the first two weeks of the survey	
5	The number of people who had been out of hospital within 12 months before the survey	
6	The type of this household: (1) The first investigation (2) The second investigation	
7	The type of this household: (1) Selected (2) Substituted	
8	The duration of investigation (minutes):	
